# Tissue-specific genes as an underutilized resource in drug discovery

**DOI:** 10.1038/s41598-019-43829-9

**Published:** 2019-05-10

**Authors:** Maria Ryaboshapkina, Mårten Hammar

**Affiliations:** Translational Science, Cardiovascular, Renal and Metabolism, IMED Biotech Unit, AstraZeneca, Gothenburg, Sweden

**Keywords:** Computational biology and bioinformatics, Drug discovery

## Abstract

Tissue-specific genes are believed to be good drug targets due to improved safety. Here we show that this intuitive notion is not reflected in phase 1 and 2 clinical trials, despite the historic success of tissue-specific targets and their 2.3-fold overrepresentation among targets of marketed non-oncology drugs. We compare properties of tissue-specific genes and drug targets. We show that tissue-specificity of the target may also be related to efficacy of the drug. The relationship may be indirect (enrichment in Mendelian disease and PTVesc genes) or direct (elevated betweenness centrality scores for tissue-specifically produced enzymes and secreted proteins). Reduced evolutionary conservation of tissue-specific genes may represent a bottleneck for drug projects, prompting development of novel models with smaller evolutionary gap to humans. We show that the opportunities to identify tissue-specific drug targets are not exhausted and discuss potential use cases for tissue-specific genes in drug research.

## Introduction

Drug development is a long and costly process. Selection of the right target is a major factor influencing the probability of success of a drug development program^1–3^. Low confidence in biological target has been linked to failures in phase 2 clinical trials due to lack of efficacy^1^ indicating that is not completely understood how to de-risk the selection of new targets. One possibility to de-risk the discovery process is to revisit gene categories that are known to have an increased probability of yielding successful drug targets. Open opportunities to discover new targets are likely not exhausted. A recent study by Oprea *et al*. indicates that only 3% of human proteins are targeted by marketed or clinical trial drugs (“Tclin”) whereas 35% have an unknown biological function and are not actively studied (“Tdark”)^4^.

In this study, we revisit tissue-specific genes. Narrow expression in one or a few tissues is considered desirable for drug targets due to reduced risk of side effects^5,6^ and such genes with narrow expression are often called ‘tissue-specific’ or ‘tissue-enriched’. Studies on microarray^7–9^ and a combination of RNA-sequencing and proteomics data^10,11^ confirm that targets of marketed drugs are biased towards tissue-specific genes. To the best of our knowledge, the first quantitative estimate was published in 2008. Dezso *et al*. demonstrated that tissue-specific genes are twice more likely to become drug targets than broadly expressed house-keeping genes^12^. Yang *et al*. confirmed a 1.7-fold higher likelihood in 2016^13^. Dezso *et al*. observed that tissue-specific genes may represent attractive drug targets due to their role in tissue biology and disease (e.g., brain-specific *GABRB2*, a receptor for the inhibitory neuromediator gamma-aminobutyric acid, is a target of sedative agents)^12^. These studies assessed tissue-specificity in healthy tissues. Their findings also extrapolate to diseased tissues because targets of marketed and phase 3 drugs are expressed in disease-relevant tissues even in the healthy state in 87% of the cases^14^. Also, an important parallel exists between tissue-specific genes and targets of marketed drugs. As first demonstrated in 2004, tissue-specific genes are enriched in Mendelian disorder genes^15^. The enrichment was confirmed by Yang *et al*. in 2016^13^. 53% targets of marketed drugs are implicated in Mendelian disorders^16^. Drugs targeting genes with a genetic link to human disease are less likely to fail in clinical trials due to lack of efficacy^1,16,17^. Thus, there may be a relationship between tissue-specificity of the target and efficacy of the drug. In fact, a recent study by Rouillard, Hurle and Agarwal concentrated on identification of omics features distinguishing targets that succeeded and failed in phase 3 trials for non-oncology diseases^18^. Phase 3 trial failures were enriched in failures due to lack of efficacy. Rouillard and colleagues limited their analysis to drugs with a single mechanism-of-action target and demonstrated that narrow expression profile of a drug target is a robust predictor of success in phase 3^18^. If we understand the relationship between tissue-specificity and efficacy and apply this knowledge to identify new, and not necessarily only tissue-specific, targets, we may ultimately reduce attrition rates in the clinic.

Here, we find that tissue-specific genes are mostly relevant for non-oncology disease indications. Application of increasingly stringent definitions of tissue-specificity leads to increasingly stronger enrichment of tissue-specific genes among marketed non-oncology drug targets. With moderately stringent definition (x = 6), we confirm a 2.3-fold enrichment among targets of non-oncology drugs and 1.8-fold enrichment in a pooled analysis for both oncology and non-oncology drug targets, which are similar to the previously published estimates^12,13^. We observe that this historic success of tissue-specific targets is not reflected in early clinical trials neither for oncology nor for non-oncology diseases, i.e., tissue-specific targets are underutilized. The limiting factor for development of tissue-specific targets may be the reduced conservation of tissue-specific genes between humans and animal models and the associated challenges in preclinical research. We find two factors, that could be related to efficacy of drugs targeting tissue-specific genes. First, we confirm enrichment in Mendelian disease genes and observe enrichment in potential disease genes with gain-of-function but not loss-of-function mechanism among tissue-specific genes. Second, we find that tissue-specific enzymes and secreted proteins have higher ability to spread perturbations in topological analysis of human protein-protein interactome.

## Results

Our results section is structured as follows. We investigate the prevalence of tissue-specific genes among targets of candidate and marketed drugs. Next, we explore properties that may explain depletion of tissue-specific genes among targets of drugs in early clinical trials and their overrepresentation among targets of marketed drugs. Finally, we highlight open opportunities to develop tissue-specific genes as drug targets.

We talk about genes as drug targets because the previous studies demonstrated enrichment in tissue-specific genes among drug targets based on mRNA expression^12,13^. We also define tissue-specificity based on RNA-sequencing data. We assume that the messenger RNAs are translated to their protein products, which, in turn, interact with the drugs. The concordance between gene expression and protein abundance is debated^19,20^, but a recent Ribo-seq study in rat suggests that 70 (heart) to 85% (liver) of transcribed mRNA are forwarded to translation^21^.

### Tissue-specific drug targets were more relevant for non-oncology indications

Tissue-specific genes constituted a small fraction of all human protein-coding genes (Supplementary Data 1). We applied nine increasingly stringent definitions of tissue-specificity x from 2 to 10 meaning at least x-fold difference in magnitude of the highest and the second highest per-tissue Z-score. For example, *SLC43A1* (https://gtexportal.org/home/gene/SLC43A1), *SCTR* (https://gtexportal.org/home/gene/SCTR) and *INS* (https://gtexportal.org/home/gene/ENSG00000254647.2) were tissue-specific for pancreas at x = 2, 6 and 10, respectively. The most liberal definition x = 2 resulted in 4,573 of 18,377 (24.9%) tissue-specific genes. 1,018 genes (5.5%) satisfied moderately stringent definition x = 6, while only 557 (3.0%) genes satisfied the most stringent definition x = 10.

**Figure 1:**
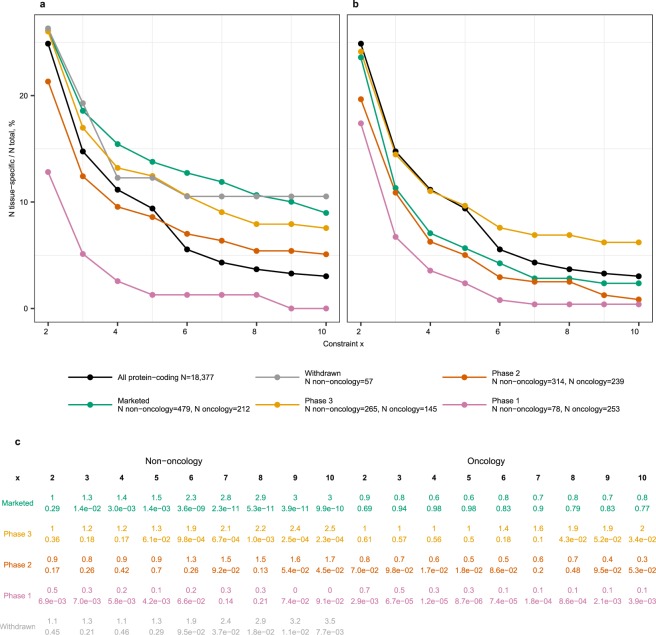
Tissue-specific genes were overrepresented among targets of phase 3 drugs and targets of marketed non-oncology drugs. Prevalence of tissue-specific genes among targets of drugs for (**a**) non-oncology and (**b**) oncology indications. Percentages of tissue-specific genes among targets of drugs in each phase of clinical development were plotted in comparison to the ‘background’ distribution among all protein coding-genes (black line). Tissue-specificity was defined at nine increasingly stringent constraints x = 2 to 10. (**c**) Fisher test p-value and fold enrichment for each gene category and each constraint x. Enrichment values >1 indicated over-representation of tissue-specific genes while values <1 indicated under-representation of tissue-specific genes. Nominal p-values < 0.05 were considered statistically significant.

If tissue-specificity was irrelevant for drug target discovery, the proportions of tissue-specific genes among drug targets would follow the ‘background’ distribution among all protein-coding genes. By contrast, we observed increasingly stronger deviations from the ‘background’ distribution with increasingly stringent definitions of tissue-specificity (Fig. 1). Targets for oncology and non-oncology disease indications were considered separately because they are selected following different discovery paradigms (e.g., different acceptability of side effects, selection of proteins harbouring cancer-specific mutations as targets etc). Targets of phase 1 drugs were significantly depleted of tissue-specific genes for both oncology and non-oncology indications even at the most liberal x = 2. The discrepancies in prevalence of tissue-specific genes between oncology and non-oncology targets started to emerge in phase 2. Enrichment in tissue-specific genes among targets of phase 3 drugs was observed for both non-oncology and oncology indications. Interestingly, the tissue-specific phase 3 oncology targets were aberrantly expressed in cancer (e.g., *GNRHR* is pituitary-specific in non-diseased state but ectopically expressed in endometrial cancers) or were targets of therapies accompanying cytostatic agents (e.g., hemoglobin as target of experimental drugs increasing tissue oxygenation to sensitize tumors to main therapy, NCT00083304). Targets of marketed non-oncology drugs were enriched in tissue-specific genes, but targets of marketed oncology drugs were depleted in tissue-specific genes. The enrichment among targets of marketed non-oncology drugs was stronger (2.3-fold at x = 6 and up to 3-fold at x = 10) than the previous estimates^12,13^, which was probably because previous studies did not make a distinction between oncology and non-oncology drug targets. Indeed, a pooled analysis for oncology and non-oncology drug targets indicated a significant 1.8-fold enrichment at moderately stringent x = 6, p-value 2e-6 (Supplementary Fig. S1). Targets of withdrawn non-oncology drugs were also enriched in tissue-specific genes. The reason for withdrawal from the market was toxicity with few exceptions like unintended use for self-poisoning (barbiturates) and lack of efficacy (drotrecogin alpha). Targets of withdrawn drugs had 95% overlap with targets of marketed drugs (57 of 60, from which only 3 were for oncology indications). Hence, withdrawal of these drugs from the market could not be uniquely attributed to their mechanism-of-action targets. For example, cholinergic nicotinic receptors *CHRNA1*, *CHRND* and *CHRNG* are targets of curare-like neuromuscular blocking agents. Rapacuronium bromide was withdrawn from the market due to adverse events while other drugs like vecuronium continue to be used.

### Tissue-specific targets of marketed drugs were less reused as targets of candidate drugs

The overlap between targets of withdrawn and marketed drugs motivated us to examine ‘recycling’ of drug targets. Target genes can reenter clinical trials when new drugs are developed for the same (e.g., generations of H_2_ histamine receptor *HRH2* blockers as anti-ulcer drugs) or a novel indication. For example, *IGF1R* is targeted by recombinant insulin growth factor 1 Mecasermin for growth failure in children (marketed agonist drug) and is evaluated as target for treatment of solid tumours (antagonist drug PL-225B in phase 1 trial NCT01779336).

**Figure 2:**
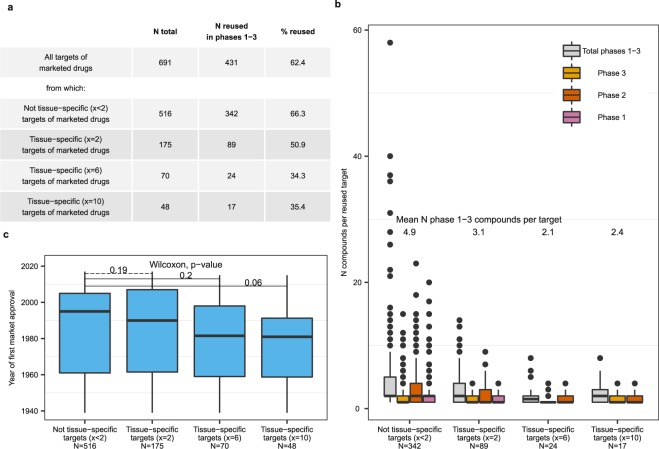
Tissue-specific targets represented less frequently reused (**a**,**b**) and older (**c**) subsets of targets of marketed drugs. a Numbers and percentages of targets of marketed drugs that were targeted by candidate drugs in clinical trials, i.e., reused. (**b**) Number of candidate drugs per re-used target. (**c**) Year of regulatory approval by FDA or another agency of the first drugs modulating tissue-specific targets compared to non-tissue-specific targets of marketed drugs. For example, carglumic acid was the first marketed drug modulating *CPS1* and it was approved in 2010.

For simplicity of presentation, we limited this and all subsequent analyses to tissue-specific genes satisfying the liberal x = 2, moderately stringent x = 6 and the most stringent x = 10 definitions of tissue-specificity. Tissue-specific targets of marketed drugs were less frequently reused by clinical trial drugs than non-tissue specific targets (Fig. 2a) and had less chemical compounds in clinical development (Fig. 2b). For example, tissue-specific targets of marketed drugs satisfying x = 6 were reused 1.9 times less frequently than non-tissue specific targets that did not satisfy any of the definitions x (66.3% vs 34.3%, Fisher test, p-value 3e-5). Furthermore, tissue-specific genes represented older subsets of drug targets (Fig. 2c), although the differences were not statistically significant.

In summary, tissue-specific targets were not actively explored in phase 1 clinical trials and tissue-specific targets of marketed drugs were less reused. We investigated possible explanations for these trends.

### Tissue-specific genes were less evolutionary conserved

Evolutionary properties may explain the reduced utilization of tissue-specific targets. Wenhua Lv *et al*. demonstrated that targets of FDA-approved drugs are more evolutionary conserved than non-target genes^22^. By contrast, **i**n 2004, Winter, Goodstasdt and Ponting investigated expression of 4,960 human genes in 27 tissues and demonstrated that tissue-specific genes are less evolutionary conserved in mice than broadly expressed genes using K_a_/K_s_ ratios^15^. To clarify, K_a_/K_s_ is the rate of nonsynonymous K_a_ to synonymous K_s_ amino acid changes in a pair of orthologs. Fractional values of K_a_/K_s_ are indicative of negative selection pressure favoring between-species conservation of an amino acid sequence. By contrast, K_a_/K_s_ values exceeding 1 may in some cases indicate that positive selection pressure has favored evolutionary divergence of orthologs, as exemplified by immune genes adapted to species-specific pathogens^23–26^.

**Figure 3:**
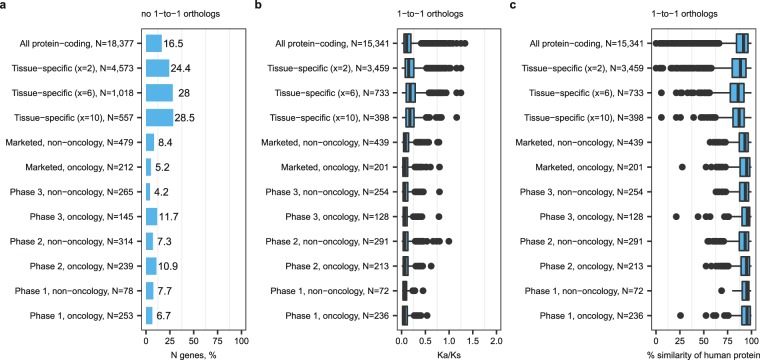
Tissue-specific genes were less evolutionary conserved in mice compared to all protein-coding genes and drug targets. (**a**) Percentages of genes without 1-to-1 orthologs in mice. 1-to-1 ortholog refers to a human gene with one unique counterpart in mouse as opposed to 1-to-many or many-to-many orthologs that arise from duplication or gene fusion events. (**b**) K_a_/K_s_ ratios for human-mouse 1-to-1 orthologs. (**c**) Sequence similarity of human protein-coding genes and their 1-to-1 orthologs in mice.

We performed analysis of evolutionary conservation on a genome-wide scale and expanded to other species. We examined percentages of human genes without 1-to-1 orthologs, K_a_/K_s_ ratios and protein sequence similarity for human protein-coding genes and their counterparts in mice and 6 other common animal model species. The analysis of K_a_/K_s_ ratios was limited to mammalian species as the evolutionary distances between human and the two non-mammalian species were too large. We confirmed that tissue-specific genes were significantly less evolutionary conserved than all protein-coding genes, in contrast to targets of not only marketed but also clinical trial drugs (Fig. 3 for mice and Supplementary Figs S2–S7 for other species, statistical analysis results for each species in Supplementary Dataset 3). Increased percentages of genes without 1-to-1 orthologs, elevated K_a_/K_s_ ratios and reduced percentages of sequence similarity for orthologs of tissue-specific genes indicated increased risk of translational gaps because the conservation of protein sequence is considered a proxy for conservation of biological function^27^ and 1-to-many or many-to-many orthologs arising through duplication tend to diverge in function with time^28,29^. FDA requires pharmacological effects and toxicological data from preclinical testing in animal models (https://www.accessdata.fda.gov/scripts/cdrh/cfdocs/cfcfr/CFRSearch.cfm?fr=312.23), so reduced evolutionary conservation of tissue-specific genes may represent a bottleneck for preclinical development and explain underrepresentation of tissue-specific genes among targets that entered phase 1 trials.

In summary, underrepresentation of tissue-specific targets in phase 1 clinical trials could be related to potential translational challenges. Despite these challenges, tissue-specific genes were enriched among targets of marketed non-oncology drugs. We hypothesized that tissue-specificity was related to efficacy and not only to safety.

### Relationship between tissue-specificity of the targets and efficacy of the drugs

#### Prevalence of disease genes

Drugs, that modulate targets with genetic evidence for a human disease, are less likely to fail in clinical trials for lack of efficacy^1,16,17^. Genetic evidence likely indirectly contributes to drug efficacy because knowledge of human genetics can help to understand the biological function of the target, find target engagement biomarkers for clinical trials and estimate dose-response curves^30^ and these factors can enhance the chances of a drug program to succeed^1,3,5,6^.

**Figure 4:**
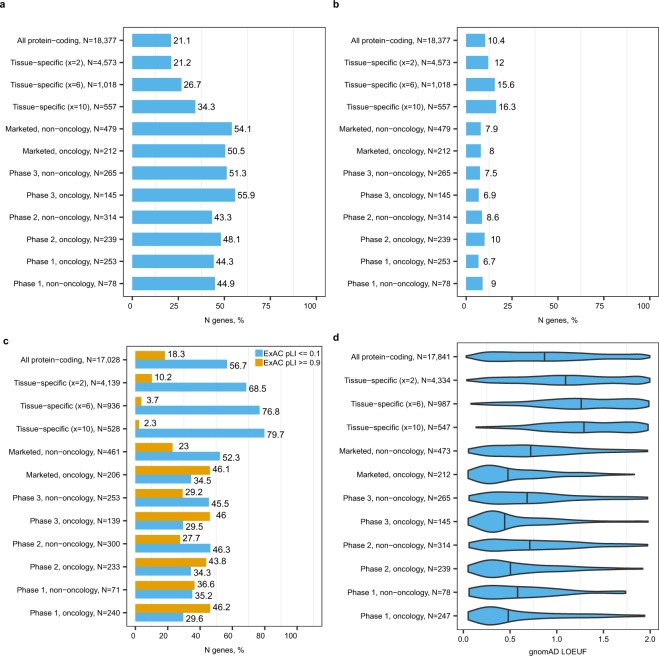
Tissue-specific genes were enriched in disease genes with potential gain-of-function mechanism but not loss-of-function mechanism. The bars show percentages of (**a**) OMIM, (**b**) PTVesc and (**c**) loss-of-function tolerant (ExAC pLI < = 0.1) and intolerant (pLI > = 0.9) genes in each gene category. Violin plots in (**d**) show loss-of-function tolerance as a continuous score according to the gnomAD consortium data. Higher values indicate higher tolerance to loss-of-function variation.

We compared the prevalence of OMIM^31^, Protein Truncating Variants *esc*aping nonsense mediated decay (PTVesc) genes^32^ and loss-of-function tolerant and intolerant^33,34^ genes among tissue-specific genes and drug targets. OMIM genes have an entry in the Online Mendelian Inheritance in Man® Morbid Map database^32^, are well-known disease genes and are likely to be explored in target discovery. Loss-of-function intolerant genes are potential disease genes that harbour significantly fewer loss-of function variants than could be expected, are subject to strong purifying selection within the human population and are largely non-redundant with OMIM^33^. PTVesc genes are an emerging class of candidate genes that can cause disease by gain-of-function mechanism. PTVesc genes are significantly depleted of genetic variants that result in nonsense-mediated-decay-escaping mRNA and production of truncated proteins with altered function (e.g., *PNPLA3* and *APOL1*)^32^. Methods for detection of PTVesc are recently developed, so PTVesc genes are unlikely to be explored to the same extent as OMIM genes. Tissue-specific genes were enriched in both OMIM and PTVesc genes (Fig. 4a and Fig. 4b, full statistical analysis in Supplementary Dataset 3). With moderately stringent definition x = 6, tissue-specific genes were 1.3-fold enriched in OMIM genes (272 of 1,018, 26.7% > 3,870 of 18,377, 21.1%, Fisher test, Bonferroni adjusted p-value 0.006) and 1.5-fold enriched in PTVesc genes (159 of 1,018, 15.6% > 1,913 of 18,377, 10.4%, Bonferroni adjusted p-value 7.4e-5). As expected, drug targets for oncology and non-oncology indications across all phases of clinical development were enriched only in OMIM genes (Fig. 4a). By contrast, the prevalence of PTVesc genes among drug targets did not significantly deviate from the overall prevalence among protein-coding genes (Fig. 4b). Tissue-specific genes were enriched in loss-of-function tolerant (ExAC consortium pLI < = 0.1) and depleted of loss-of-function intolerant (ExAC pLI > = 0.9) genes compared to all protein-coding genes (Fig. 4c, full statistical analysis in Supplementary Dataset 3). By contrast, targets of oncology drugs were enriched in loss-of-function intolerant and depleted of tolerant genes. Targets of marketed non-oncology drugs had comparable prevalence of loss-of-function tolerant and intolerant genes compared to all protein-coding genes. We also analyzed a continuous loss-of-function tolerance metric LOEUF published by the gnomAD consortium^34^ and confirmed the results obtained with ExAC consortium data (Fig. 4d). Loss-of-function tolerance metrics for tissue-specific genes were more similar to non-oncology that oncology drug targets and confirmed that tissue-specific genes were more likely to become targets for non-oncology drugs. Also, increased tolerance of tissue-specific genes to loss-of-function variation could have implications for strategies to discover disease phenotypes associated with tissue-specific genes. Future research efforts need to consider early insights from the ExAC consortium flagship paper indicating that the degree of loss-of-function tolerance has an impact on the probability that the gene is detected in a GWAS study or has eQTLs and that recessive disease genes tend to be more loss-of-function tolerant than all human genes^33^.

In total, 386 of 1,018 (37.9%) tissue-specific genes at x = 6, 1,400 of 4,573 (30.6%) at x = 2 and 250 of 557 (44.9%) at x = 10 were OMIM genes or PTVesc genes or both. For comparison, 5,362 of 18,377 (29.2%) protein-coding genes were OMIM or/and PTVesc genes. Thus, tissue-specific genes were more likely to provide necessary information for development of efficacious drugs through human genetics than protein-coding genes overall.

#### Network analysis

The ability to spread perturbations and cause phenotypic changes is a key property of drug targets, which is reflected by topological properties in protein-protein interaction (PPI) networks^35^. We performed network analysis on STRING v10.5^36^ (Supplementary Data 2) because tissue-specific proteins are well represented in this data base^37^. We investigated the sources of supporting evidence for PPIs (Supplementary Fig. S8). Tissue-specific genes did not markedly differ from all protein-coding genes in this respect. The only potential source of bias for network analysis was the amount of publications linked to a specific protein (Kendall tau b = 0.31). The calculations were performed on the largest connected component including 19,574 proteins and 5,676,527 PPIs. The unweighted network diameter was 6. We evaluated 5 centrality scores (illustrated in Supplementary Fig. S9). We included three control gene sets with known ability to affect phenotype. Essential genes^38^ and OMIM genes served as positive controls. Genes with rare homozygous loss-of-function rhLOF variants and without clinical manifestation or impact on medication prescription rate served as negative no-phenotype controls (British-Pakistani, ExAC and Icelandic individuals, Suppl. Table 2 from^39^).

Topological properties of the proteins accurately reflected their ability to spread perturbations through the network as indicated by low centrality scores for negative controls rhLOF and high centrality scores for positive control genes (Supplementary Fig. 10). Interestingly, PTVesc genes, that we expected to behave similar to OMIM positive controls, had comparable or slightly lower median centrality scores than all protein-coding genes suggesting that some aspects of signal propagation may not be captured by PPIs. Elevated betweenness centrality was the only topological property that could distinguish tissue-specific genes from all protein-coding genes (Table 1). The trend was nominally significant at x = 6 and passed the correction for multiple testing at x = 2 and x = 10. Specifically, enzymes and secreted proteins, that were expressed in a tissue-specific manner, had higher betweenness centrality scores than other tissue-specific genes (Table 2). Our results were consistent with the previous study on regulatory networks, in which Sonawane *et al*. applied a less stringent definition of tissue-specificity and found that tissue-specific genes serve as “bottlenecks” on signaling paths^40^.

**Table 1:** Tissue-specific genes had elevated betweenness centrality scores compared to all protein-coding genes and negative controls (rhLOF) in STRING v10.5.

Group	Median (IQR)	Nominal p	Bonferroni p
All proteins, N = 19,574	14, (0–19,548)	Reference	Reference
Tissue-specific (x = 2), N = 4,474	30, (0–19,534.7)	1.3e-06	1.9e-05
Tissue-specific (x = 6), N = 1,004	32.1, (0–19,536.3)	7.3e-03	0.11
Tissue-specific (x = 10), N = 553	38.6, (1–19,806.7)	2.5e-04	3.7e-03
Essential, N = 1,713	29,414.2, (40–130,570)	8.9e-179	1.3e-177
OMIM, N = 3,844	14,754.6, (7–59,036.2)	2.6e-169	3.9e-168
PTVesc, N = 1,912	13, (0–19,012.8)	0.17	1
rhLOF, N = 107	8, (0–2,241.5)	0.20	1
Marketed, oncology, N = 211	48,734.4, (11,077.1–201,844.9)	2.0e-46	3.0e-45
Marketed, non-oncology, N = 476	1,326, (13–42,544)	9.4e-23	1.4e-21
Phase 3, oncology, N = 145	70,125.7, (15,160.1–36,5211.2)	6.5e-34	9.8e-33
Phase 3, non-oncology, N = 265	8,555.3, (38.2–59,080.5)	5.3e-21	8.0e-20
Phase 2, oncology, N = 239	58,807, (595.9–375,702)	9.4e-46	1.4e-44
Phase 2, non-oncology, N = 314	18,58.1, (8.8–51,355.6)	4.4e-15	6.6e-14
Phase 1, oncology, N = 253	51,848.7, (5,140.7–222,555.3)	6.8e-46	1.0e-44
Phase 1, non-oncology, N = 78	12,652.2, (51.5–58,423.5)	2.9e-07	4.3e-06

IQR stands for interquartile range. P-values are from two tailed Mann-Whitney U test between the gene categories and all protein-coding genes (marked as ‘Reference’).

**Table 2:** Betweenness centrality scores for tissue-specific genes were elevated due to enzymes and genes encoding secreted proteins.

Group	Median (IQR)	Mann-Whitney U, p	Bonferroni p
**Tissue-specific genes (x = 2):**			
Secreted and enzyme (N = 71)	220.8 (3.1–57,712.4)	2.7e-03	1.3e-02
Secreted (N = 584)	47.4 (1–19,911.5)	2.4e-04	1.2e-03
Enzyme (N = 363)	10,147.6 (10.4–5,6871.6)	3.9e-22	1.9e-21
Transporter (N = 151)	21.8 (1–240.1)	0.40	1
Transcription factor (N = 273)	12 (0–19,366.8)	0.28	1
Neither of the above (N = 3,032)	21.8 (0–19,099.1)	Reference	Reference
**Tissue-specific genes (x = 6):**			
Secreted and enzyme (N = 42)	122.7 (1.6–40,206.9)	5.2e-02	0.26
Secreted (N = 252)	236.4 (2–2,7831.9)	7.0e-06	3.5e-05
Enzyme (N = 72)	7,294 (39-5,1895.2)	2.3e-08	1.1e-07
Transporter (N = 57)	28 (3–292.8)	0.44	1
Transcription factor (N = 48)	13 (0–19,343.1)	0.81	1
Neither of the above (N = 533)	18 (0–3,438.1)	Reference	Reference
**Tissue-specific (x = 10):**			
Secreted and enzyme (N = 34)	192.9 (1.6–40,206.9)	7.0e-02	0.35
Secreted (N = 186)	105 (2–35,507.9)	2.6e-03	1.3e-02
Enzyme (N = 54)	8,318.3 (54.9–60,946.2)	3.0e-06	1.5e-05
Transporter (N = 35)	31 (7.8–9,919.4)	0.18	0.91
Transcription factor (N = 25)	14 (1–19,570)	0.63	1
Neither of the above (N = 219)	13 (0–10,240.7)	Reference	Reference

### Open opportunities

**Figure 5:**
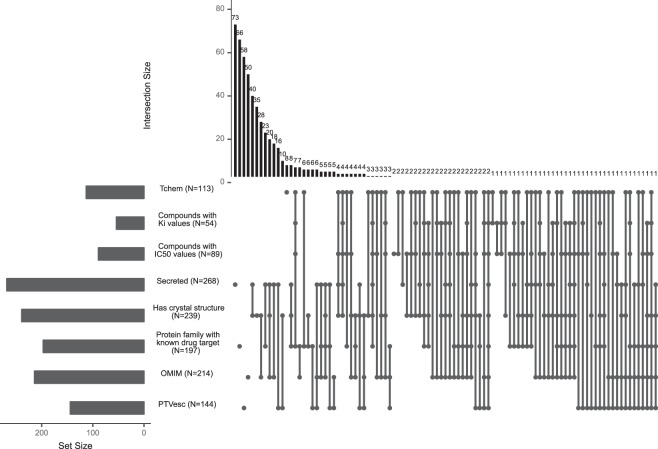
Tissue-specific genes (x = 6) that were not yet explored as targets of marketed or clinical trial drugs but were potentially druggable or had human genetic evidence. Dots indicate overlapping sets, while bars on the top indicate overlap size. For example, the fifth column indicates that 40 secreted proteins had known crystal structures and did not have other indications of druggability. Proteins classified as Tchem in the TCRD data base have potent compounds with binding affinities in the nanomolar (G-protein coupled receptors, nuclear hormone receptors, kinases) or lower micromolar range (ion channels and other target categories). Such chemical compounds can be optimized and transitioned to clinical trials. Secreted proteins may be amenable to antibody therapies. Other tissue-specific genes have some knowledge around them to start a chemical development program. Chemical compounds with binding activities in ChEMBL v23, possibly less potent than the criteria used to define Tchem, offer hints to infer structure-activity relationships, to discover and optimize a lead compound. Known crystal structure may be used to identify binding pockets and design binding molecules. Known drug targets in the same protein family may aid chemical discovery through sequence similarity and homology modelling.

We estimated the numbers of tissue-specific genes with some initial indication of druggability and human genetic evidence through OMIM and PTVesc (Fig. 5 for x = 6, Supplementary Fig. S11 for x = 2 and Supplementary Fig. S12 for x = 10) and observed that the opportunities were not exhausted. In total, only 100 of 1,018 (9.8%) tissue-specific gene satisfying the moderately stringent definition x = 6 were explored as targets of marketed or clinical trial drugs. 284 of the remaining 918 (30.9%) tissue-specific genes were classified as Tdark in the TCRD data base^41^, i.e., were poorly researched with unknown biological function. 529 of 918 (57.6%) showed some indication of druggability by small molecule or antibody approaches and 211 of 918 (22.9%) had both indications of druggability and human genetic evidence (Fig. 5). The definition of druggability constantly expands, and targets that cannot be modulated with small molecules or antibodies may be druggable with other approaches in the future.

## Discussion

The limitation of our study is that we conducted a retrospective analysis of tissue-specific genes compared to drug targets. Targets of phase 1 drugs reflect the most recent research. By contrast, targets of marketed drugs have undergone at least a decade in preclinical and clinical development and reflect older research. Theoretically, overrepresentation of tissue-specific targets on the market and depletion in phase 1 could reflect a historic shift in target selection paradigms. However, Rouillard and colleagues^18^ studied phase 3 drugs (projects with comparable “age”) and demonstrated that drugs modulating tissue-specific targets are more likely to succeed in phase 3 and gain regulatory approval. Hence, the data presented in Fig. 1 and Supplementary Fig. S1 do not merely represent a historic trend and we are justified to state that drugs modulating tissue-specific targets are indeed more likely to progress in the clinic. Also, we have based the analysis on the GTEx tissue panel. We selected this tissue panel because it both includes large number of tissues, which are necessary to make robust conclusions about tissue-specificity, and, to the best of our knowledge, the largest sample size per tissue, which is crucial for robust calculation of Z-scores because they are derived from mean expression values. Some tissues (e.g., bone marrow) and disease samples are not included in GTEx. We recommend to use databases such as TiGER^42^, TiSGeD^43^, VeryGene^44^ and TissGDB^45^ if tissue-specificity in disease or tissues absent in GTEx represent the focus of reader’s research.

**Figure 6:**
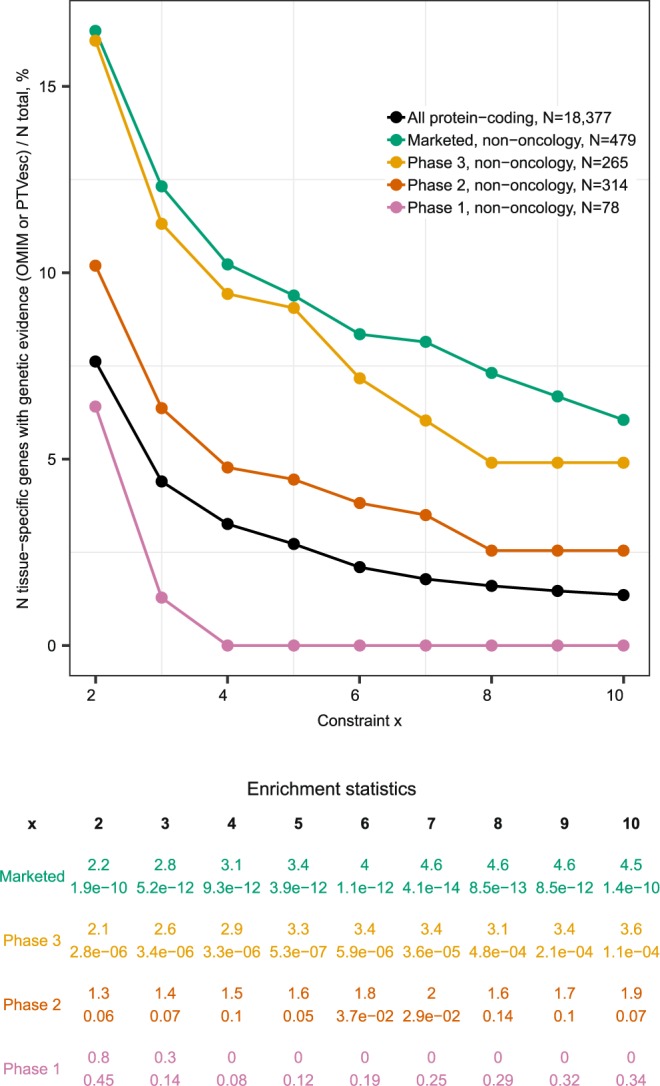
Combining tissue-specificity with genetic evidence may represent an effective de-risking strategy for non-oncology drug targets. The graph shows prevalence of genes that are both tissue-specific at each x and have an OMIM Morbid Map entry or are PTVesc among drug targets compared to all protein-coding genes. The table below contains fold enrichment and p-values. Enrichment <1 indicates depletion in tissue-specific genes with human genetic evidence.

In contrast to previous works reporting increased tissue-specificity of drug targets^7–13^, we applied nine definitions of tissue-specificity of varying stringency and considered targets of oncology and non-oncology dugs separately. This analysis approach enabled us to establish that tissue-specific genes are mostly relevant for non-oncology disease indications. We observed that increasingly stringent definitions of tissue-specificity lead to increasingly stronger overrepresentation of tissue-specific genes among targets of marketed non-oncology drugs with statistically significant enrichment starting from x = 3. The statistical significance was reached staring from x = 6 in a pooled analysis for both oncology and non-oncology indications. We found that a substantial proportion of tissue-specific genes have genetic evidence that could facilitate drug development. In fact, combining simple criteria of tissue-specificity with genetic evidence for target prioritization could be a potentially effective de-risking strategy for non-oncology indications (Fig. 6) but this strategy should also be validated in prospective studies.

We conclude that tissue-specific genes are a promising source for target discovery. Feasibility-related hurdles in development of tissue-specific targets could be circumvented by development of *in vivo* models (e.g., humanized mice, human IPS-derived organoids transplanted in animal models). In addition, genes that are currently considered undruggable could be amenable to novel therapeutic approaches. For example, tissue-specific genes causing monogenic Mendelian diseases are potential targets for genome editing with CRISPR/Cas9 (e.g., *SERPINA1* in alpha-1 antitrypsin deficiency^46^). Tissue-specific genes could also be used for targeted delivery (e.g., oncolytic viral therapies like urothelium-specific adenovirus CG8840 with bladder-specific *UPK2* promoter for bladder cancer^47^ or N-acetylgalactosamine (GalNAc)-conjugated antisense oligonucleotide drugs binding to *ASGR1* for targeted delivery to hepatocytes^48^). Also, tissue-specific genes with protein products entering the bloodstream may find applications as biomarkers (*KLK3* in prostate cancer) or replacement therapies (e.g., insulin in type 1 diabetes).

## Materials and Methods

### Gene expression

Gene-level RPKM values were downloaded from The Genotype-Tissue Expression Consortium^49^ (https://gtexportal.org/home/, release 6). The per-tissue mean RPKM for each gene was subjected to Z-transformation across tissues and then to a second Z-transformation across genes to bring all Z-scores to the same scale. We identified 18,377 protein-coding genes with HGNC approved gene symbol^50^. The non-alternative loci data set was obtained from the HGNC Database (www.genenames.org, 30.08.2017).

### Definitions of tissue-specificity

We applied peak-based definitions of tissue-specificity. The peak x was defined as the difference between the highest and the second highest per-tissue Z-scores for each gene. Nine increasingly stringent definitions were applied: Z_second largest_ <1/x * Z_max_, where Z_max_ denoted the Z-score in the tissue with the highest expression, Z_second largest_ denoted the Z-score in tissue with the second highest expression and x was an integer from 2 to 10. So, x = 2 indicated at least two-fold difference in Z-scores, i.e., difference by at least 2 standard deviations from the overall mean. Such definitions allowed genes to be expressed in multiple tissues, as long as the expression in the tissue with the maximal expression was distinctly higher than in all other tissues. Since the definitions were based on the inequality Z_second largest_ <1/x * Z_max_, stringent x automatically implied that all more liberal x were also satisfied while the opposite statement was not automatically true. For example, secretin receptor was considered pancreas-specific at x = 6 and accordingly at all more liberal constraints x 2 to 5 but did not satisfy more stringent constraints x 7 to 10 (https://gtexportal.org/home/gene/SCTR). Also, by definition, genes satisfying a more stringent x are a subset of genes satisfying more liberal x (e.g., x = 10 are a subset of genes satisfying x = 9, which in turn are a subset of genes satisfying x = 8 etc).

### Drug targets

Mechanism-of-action targets of marketed and clinical trial drugs, disease indications and year of first approval for marketed drugs were extracted from ChEMBL version 23^51^. Drugs were classified as phase 1, 2, 3 or marketed drugs based on the maximal phase they reached in clinical trials. Disease indications were mapped to Disease Ontology^52^. Proteins were classified as oncology or non-oncology targets based on parent terms in Disease Ontology. If a protein was targeted by at least 1 oncology drug, it was considered an oncology target.

### Meta-data

Example compounds with exact K_i_ or IC_50_ activity values against human proteins, measured in assays with direct interaction and the highest confidence score = 9, were retrieved from ChEMBL v23^51^. Mapping from ENSEMBL identifiers to PDB were obtained from GENECODE consortium^53^ version 27. Mapping to enzyme EC numbers, Uniprot and NCBI Gene (Entrez) identifiers were extracted from the HGNC non-alternative loci data set^50^. Target Development Level (TDL) was retrieved from TCRD version 4.6.2^41^. Subcellular localization and protein family information were obtained from UniProt/SwissProt^54^. Probabilities of being loss-of-function intolerant (pLI) were retrieved from Supplementary Data of the ExAC consortium flagship publication^33^. Associations with Mendelian diseases were retrieved from OMIM Morbid Map^31^ (copyright John Hopkins University, AstraZeneca purchased license JHU agreement number A30699 and reference number C03746). We included only binary indicator variables (has/does not have an entry in the Morbid Map). Number of PubMed-indexed articles linked to each gene was retrieved from NCBI Gene^55^ https://www.ncbi.nlm.nih.gov/gene/ on the 02.01.2018. Human to mouse orthologs, K_a_/K_s_ ratios and percentages of sequence identity and similarity were extracted from ENSEMBL Compara^56^ version 95. The lists of essential genes^38^, PTVesc^32^ and rhLOF^39^ genes and human transcription factors^57^ were obtained from supplementary data of the respective publications. Biological function of the genes was described according to the NCBI Gene/RefSeq summary^58^ unless explicitly indicated otherwise.

### Network analysis

Human protein-protein interaction network was downloaded from STRING v 10.5 (file 9606.protein.links.detailed.v10.5.tsv)^36^. Topological properties were calculated with igraph^59^ version 1.2.1. Weighted k-shell decomposition was computed as described in^60^. Combined evidence scores were used as edge weights for strength, eigenvector centrality and k-shell calculations, i.e., the overall ‘influence’ of a node was proportional to the number of its neighbors combined with confidence in its PPIs. Edge weights were taken as (1 – combined evidence score) for centrality measures based on shortest paths, i.e., shortest paths were the ‘least uncertainty paths’.

### Statistical analysis

We applied Fisher exact test for count data because sample sizes were small in some instances (e.g., 3 tissue-specific targets in phase 1 at x = 6) and to be consistent in other analyses. Mann-Whitney U test was used to test differences between groups for continuous variables. Wilcoxon test with explicit handling of tied values in exactRankTests^61^ version 0.8–29 was used to test differences in year of first approval. Tests for enrichment or depletion were one-tailed, other tests were two-tailed. Bonferroni correction for multiple testing was applied as appropriate. P-values < 0.05 were considered significant. Statistical analyses were summarized in Supplementary Dataset 3. Figures were generated with ggplot2^62^ version 3.0.0, viridis^63^ version 0.5.1 and UpSetR^64^ version 1.3.3. Analyses were performed in R^65^ version 3.4.1.

## Supplementary information

Dataset 3

Supplementary Figures

Dataset 1

Dataset 2

## Data Availability

All data, that were generated in this study, are provided as Supplementary data sets. Annotated Z-score tables for protein-coding genes including the tissue-specific gene and drug target subsets are provided in Supplementary Data 1. Network topology properties are provided in Supplementary Data 2. Columns, that were used as input data for figures, are labelled within each supplementary data set. Summary-level data behind the figures are included in the Supplementary Dataset 3. Source data for evolutionary conservation can be retrieved directly from Ensembl Compara^56^ v 95. gnomAD consortium data can be retrieved directly from https://gnomad.broadinstitute.org/.
