# Supplementary material for: Tissue-specific genes as an underutilized resource in drug discovery
Source: Sci Rep. 2019 May 10;9:7233. doi: 10.1038/s41598-019-43829-9 (PMC6510781; doi:10.1038/s41598-019-43829-9)
Supplement: Supplementary file 2 — Supplementary Figures [file 41598_2019_43829_MOESM2_ESM.pdf]

Supplementary Figures

Tissue-specific genes as an underutilized resource in drug discovery

Maria Ryaboshapkina MSc[1,*], Mårten Hammar PhD[1]

[1]Translational Science, Cardiovascular, Renal and Metabolism, IMED Biotech Unit, AstraZeneca, Gothenburg, Sweden

*maria.ryaboshapkina@astrazeneca.com

AstraZeneca

Pepparedsleden 1

431 50 Mölndal

Sweden

a

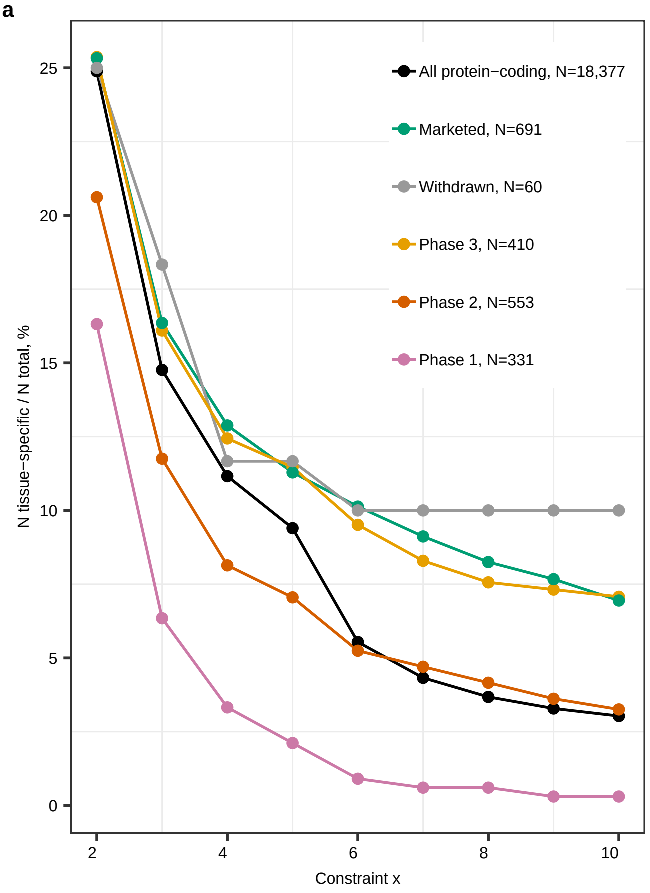

b

x	2	3	4	5	6	7	8	9	10
Marketed	1<br>0.41	1.1<br>0.14	1.2<br>0.09	1.2<br>0.06	1.8<br>2e−06	2.1<br>8e−08	2.2<br>5e−08	2.3<br>5e−08	2.3<br>3e−07
Phase 3	1<br>0.43	1.1<br>0.24	1.1<br>0.23	1.2<br>0.09	1.7<br>0.001	1.9<br>3e−04	2.1<br>2e−04	2.2<br>6e−05	2.4<br>4e−05
Phase 2	0.8<br>0.02	0.8<br>0.05	0.7<br>0.03	0.8<br>0.06	0.9<br>0.85	1.1<br>0.67	1.1<br>0.57	1.1<br>0.63	1.1<br>0.71
Phase 1	0.7<br>1e−04	0.4<br>2e−06	0.3<br>2e−07	0.2<br>1e−07	0.2<br>1e−05	0.1<br>6e−05	0.2<br>4e−04	0.1<br>2e−04	0.1<br>5e−04
Withdrawn	1<br>0.54	1.2<br>0.27	1.1<br>0.51	1.3<br>0.33	1.8<br>0.11	2.3<br>0.05	2.7<br>0.02	3<br>0.01	3.3<br>0.01

Supplementary Figure 1. Prevalence of tissue-specific targets increased from phase 1 to the market. a Percentages of tissue-specific genes among targets of drugs in each phase of clinical development were plotted in comparison to the "background" distribution among all protein coding-genes (black line). Tissue-specificity was defined at nine increasingly stringent constraints $x = 2$ to 10. **b** Enrichment statistic and Fisher test p-value for each gene category. Enrichment <1 indicates that tissue-specific genes were underrepresented in the gene category.

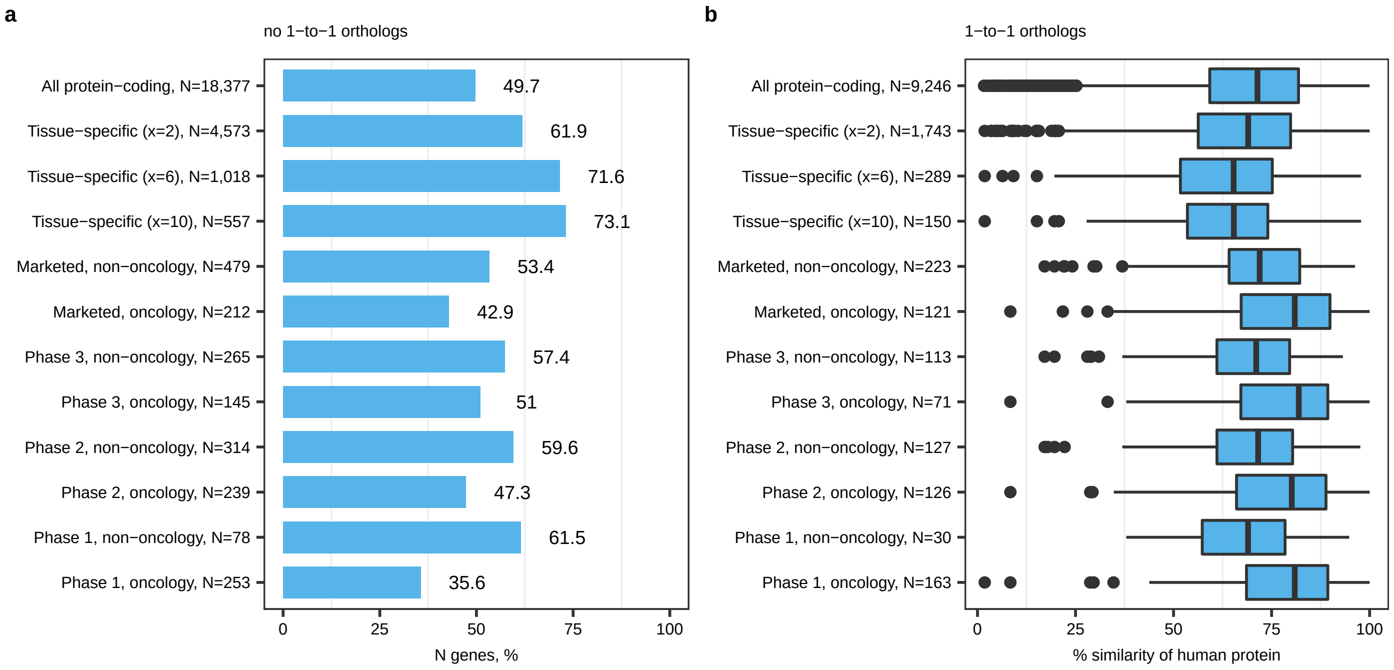

Supplementary Figure 2. Tissue-specic genes were less conserved in Danio rerio compared to all protein-coding genes and drug targets. a Percentages of genes without 1-to-1 orthologs in zebrafish. **b** Sequence similarity of human protein-coding genes and their 1-to-1 orthologs in zebrafish.

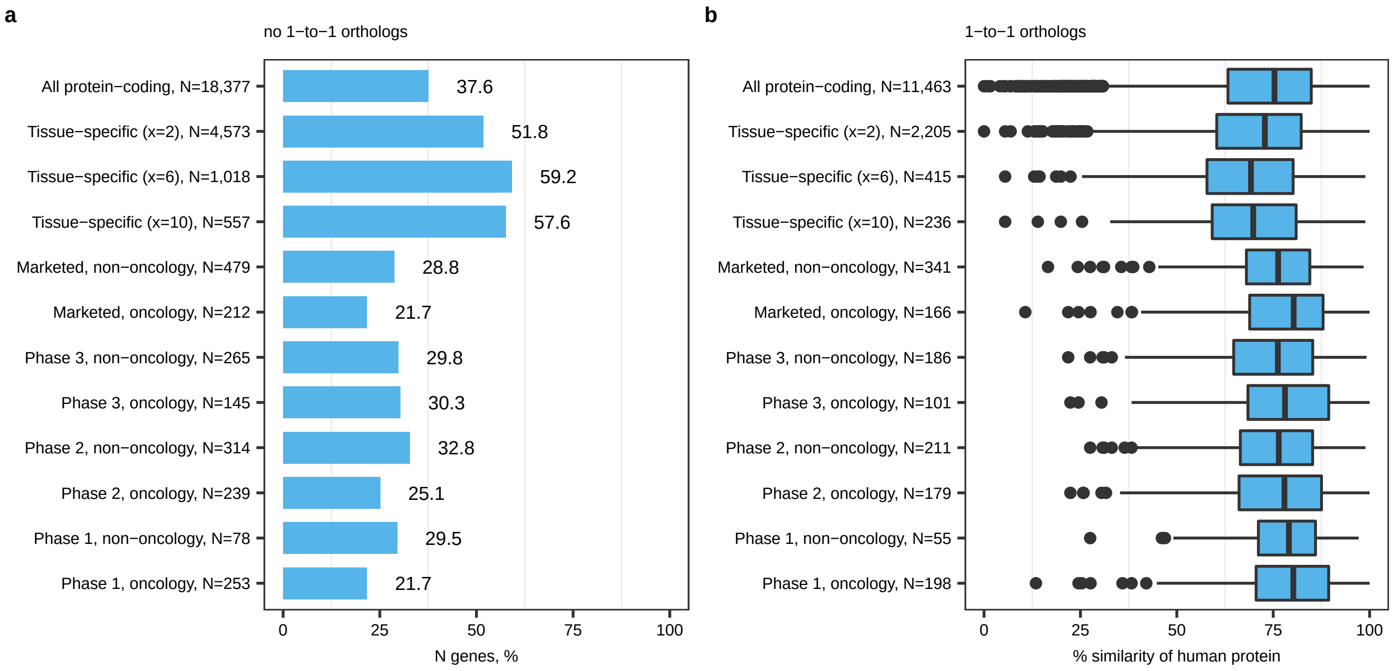

Supplementary Figure 3. Tissue-specic genes were less conserved in Xenopus tropicalis compared to all protein-coding genes and drug targets. a Percentages of genes without 1-to-1 orthologs in clawed frog. **b** Sequence similarity of human protein-coding genes and their 1-to-1 orthologs in clawed frog.

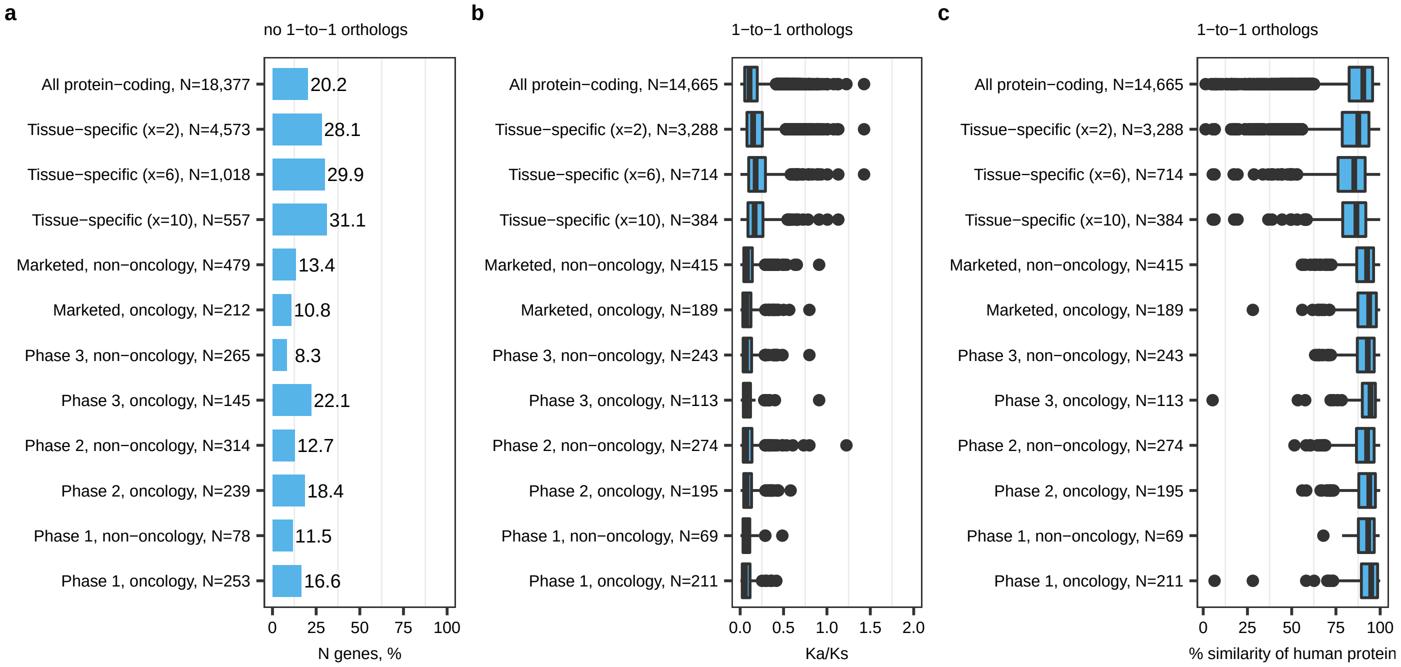

Supplementary Figure 4. Tissue-specic genes were less conserved in Rattus norvegicus compared to all protein-coding genes and drug targets. a Percentages of genes without 1-to-1 orthologs in rat. **b** Ka/Ks ratios for human-rat 1-to-1 orthologs. **c** Sequence similarity of human protein-coding genes and their 1-to-1 orthologs in rat.

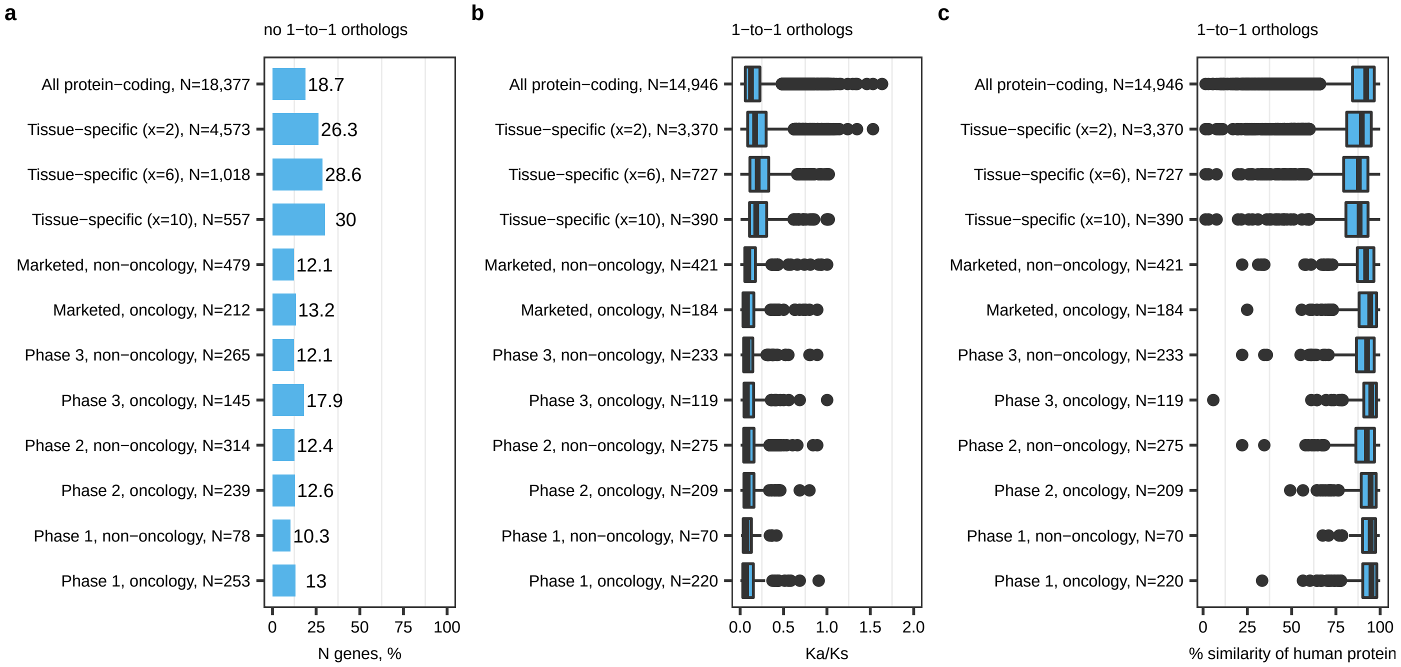

Supplementary Figure 5. Tissue-specic genes were less conserved in Canis lupus familiaris compared to all protein-coding genes and drug targets. a Percentages of genes without 1-to-1 orthologs in dog. **b** Ka/Ks ratios for human-dog 1-to-1 orthologs. **c** Sequence similarity of human protein-coding genes and their 1-to-1 orthologs in dog.

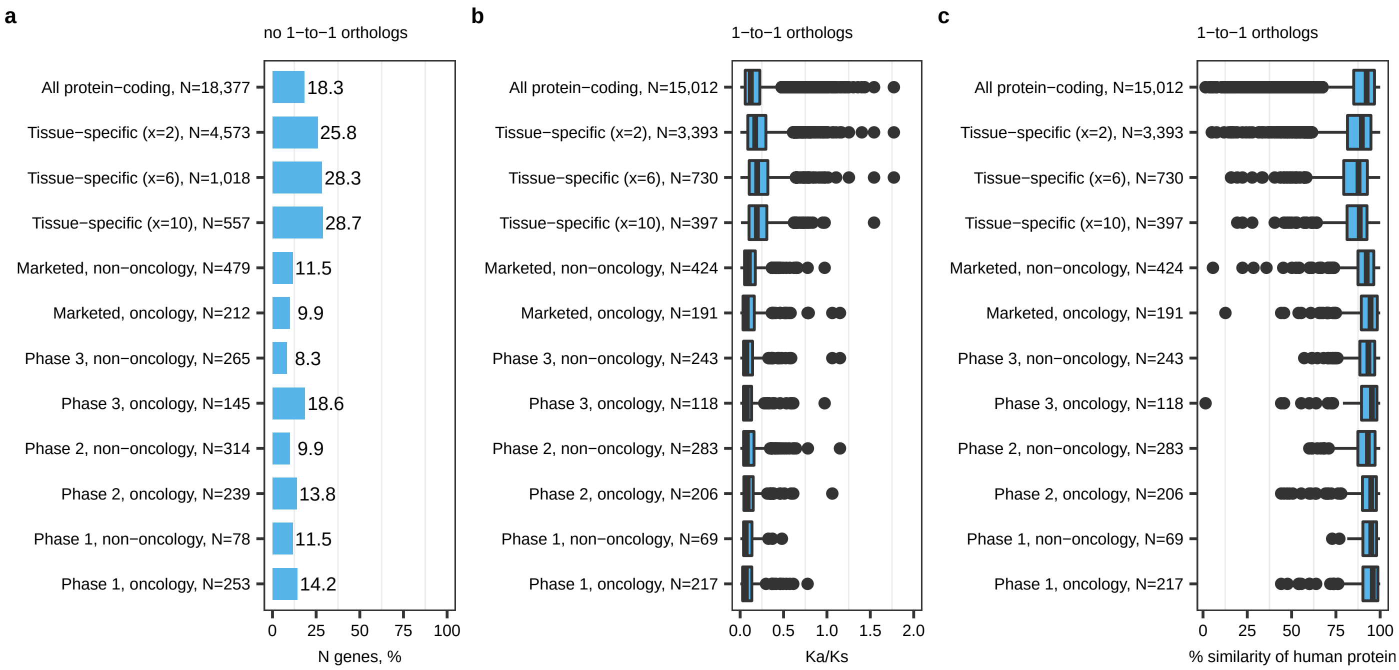

Supplementary Figure 6. Tissue-specic genes were less conserved in Sus scrofa compared to all protein-coding genes and drug targets. **a** Percentages of genes without 1-to-1 orthologs in pig. **b** Ka/Ks ratios for human-pig 1-to-1 orthologs. **c** Sequence similarity of human protein-coding genes and their 1-to-1 orthologs in pig.

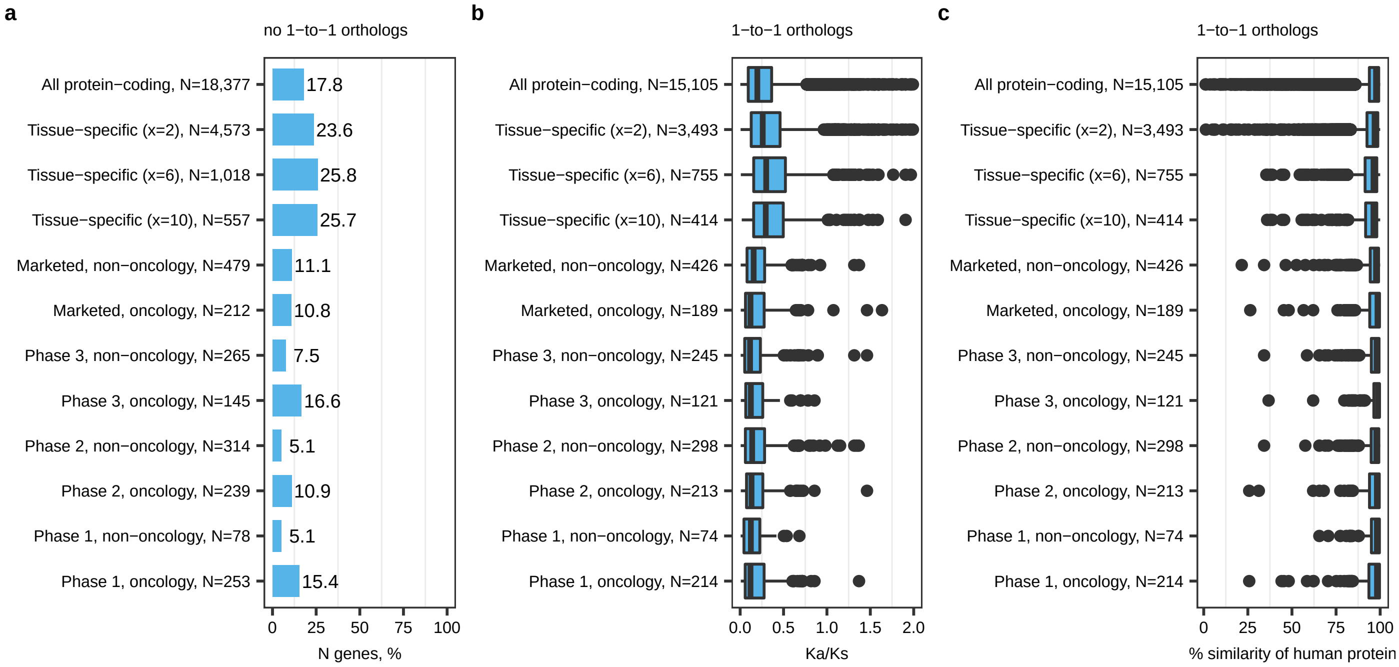

Supplementary Figure 7. Tissue-specic genes were less conserved in Macaca mulatta compared to all protein-coding genes and drug targets. **a** Percentages of genes without 1-to-1 orthologs in rhesus monkey. **b** Ka/Ks ratios for human-rhesus monkey 1-to-1 orthologs. **c** Sequence similarity of human protein-coding genes and their 1-to-1 orthologs in rhesus monkey.

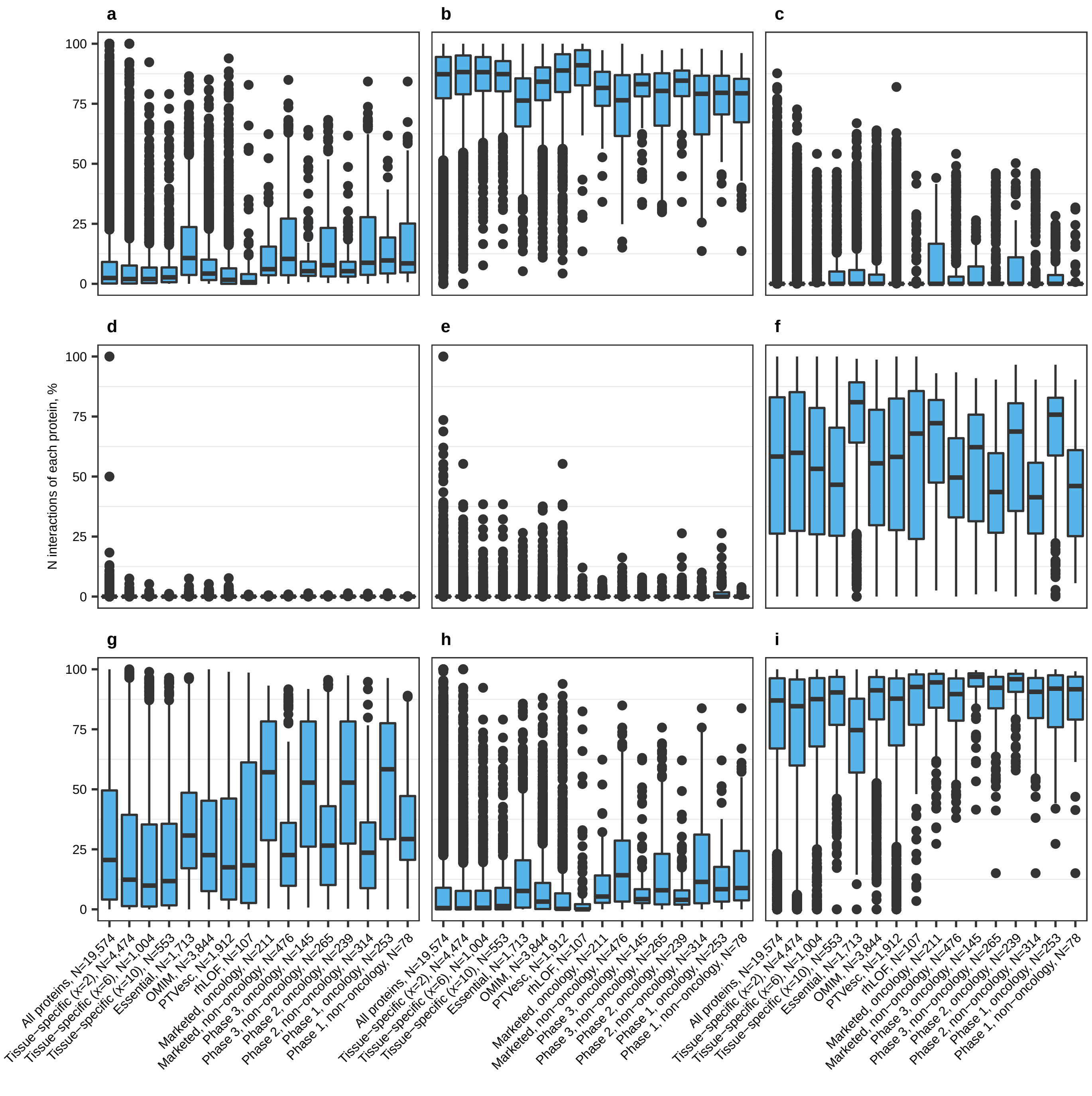

Supplementary Figure 8. Properties of protein-protein interactions (PPIs) in STRING v 10.5. PPIs with support from each evidence channel are plotted along y axis as percentage from the total number of PPIs of each protein. **a** High-confidence PPIs with combined confidence score ≥ 0.7. **b** Low-confidence PPIs with combined confidence scores <0.4. **c** PPIs with support from the neighbourhood evidence channel. **d** PPIs with support from the fusion evidence channel. **e** PPIs with support from the co-occurrence evidence channel. **f** PPIs with support from the co-expression evidence channel. **g** PPIs with support from the experimental evidence channel. **h** PPIs with support from pathway databases. **i** PPIs with support from text mining.

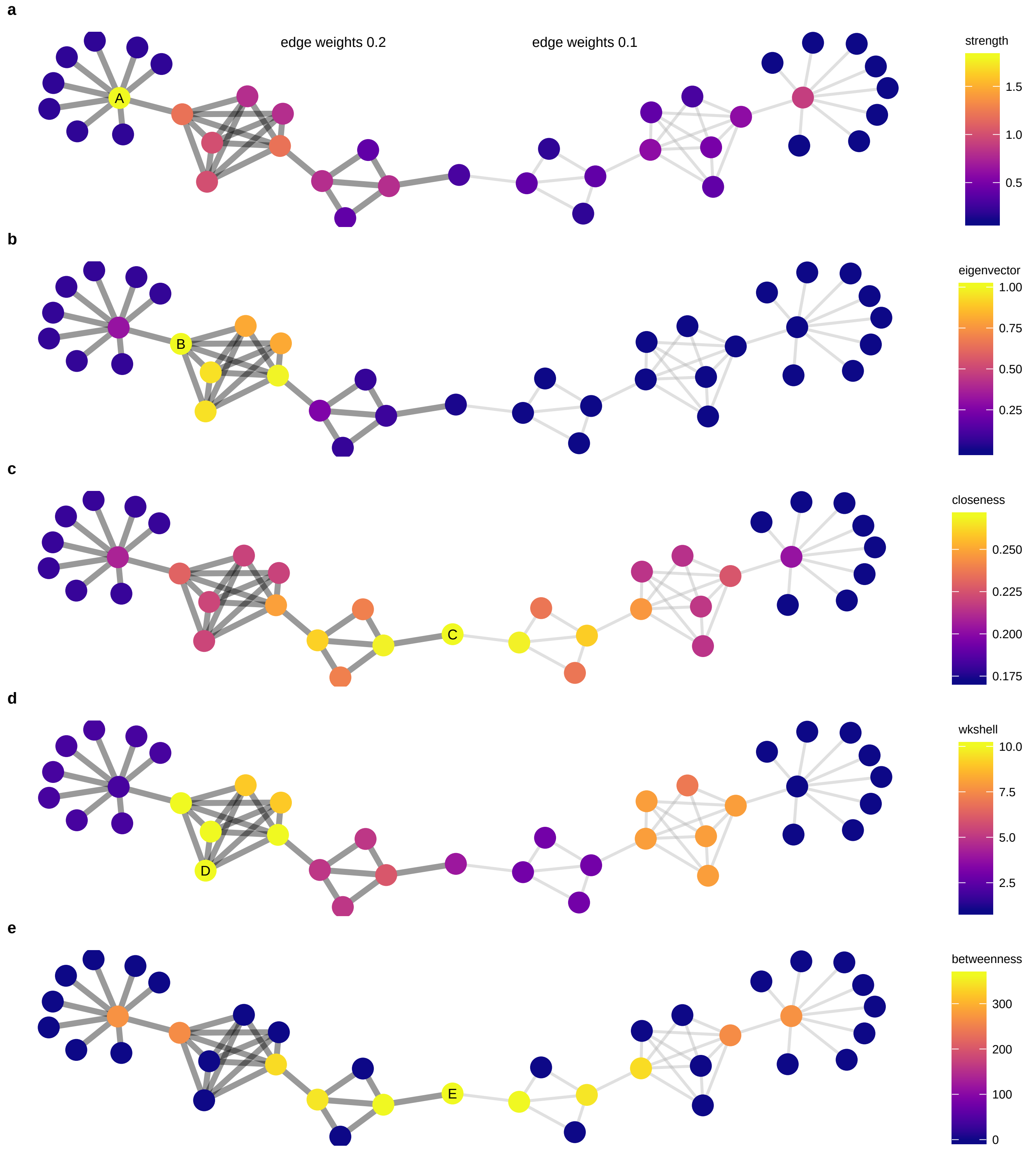

Supplementary Figure 9. Centrality scores on an example network. The example network has two symmetric parts with different edge weights to illustrate their impact on the centrality scores. Nodes with the highest values for each score are labelled A-E. **a** Strength. Strength is the sum of edge weights of a node. Nodes have high strength if they have many neighbours or interactions with high weights. **b** Eigenvector centrality. Eigenvector centrality is the dominant eigenvector of the adjacency matrix. Nodes with high strength, that are connected to other high-strength nodes, have high eigenvector centrality. **c** Closeness centrality. Closeness centrality is the inverse of the sum of shortest paths lengths to all other nodes. The node with the shortest distance to all other nodes has the highest closeness centrality. **d** Weighted k shell. Unweighted k shell is computed by recursively removing nodes with $k = 1$, 2, 3 etc. neighbours. Nodes with high k shell are in the center of the network, whereas nodes with low k shell are in the periphery. Weighted k shell allows for a more granular division of the network into shells as edge weights are incorporated into the calculations. **e** Betweenness centrality. Betweenness centrality reflects the fraction of shortest paths between all nodes that pass through a given node. Nodes with high betweenness centrality are important for communication between elements of the network.

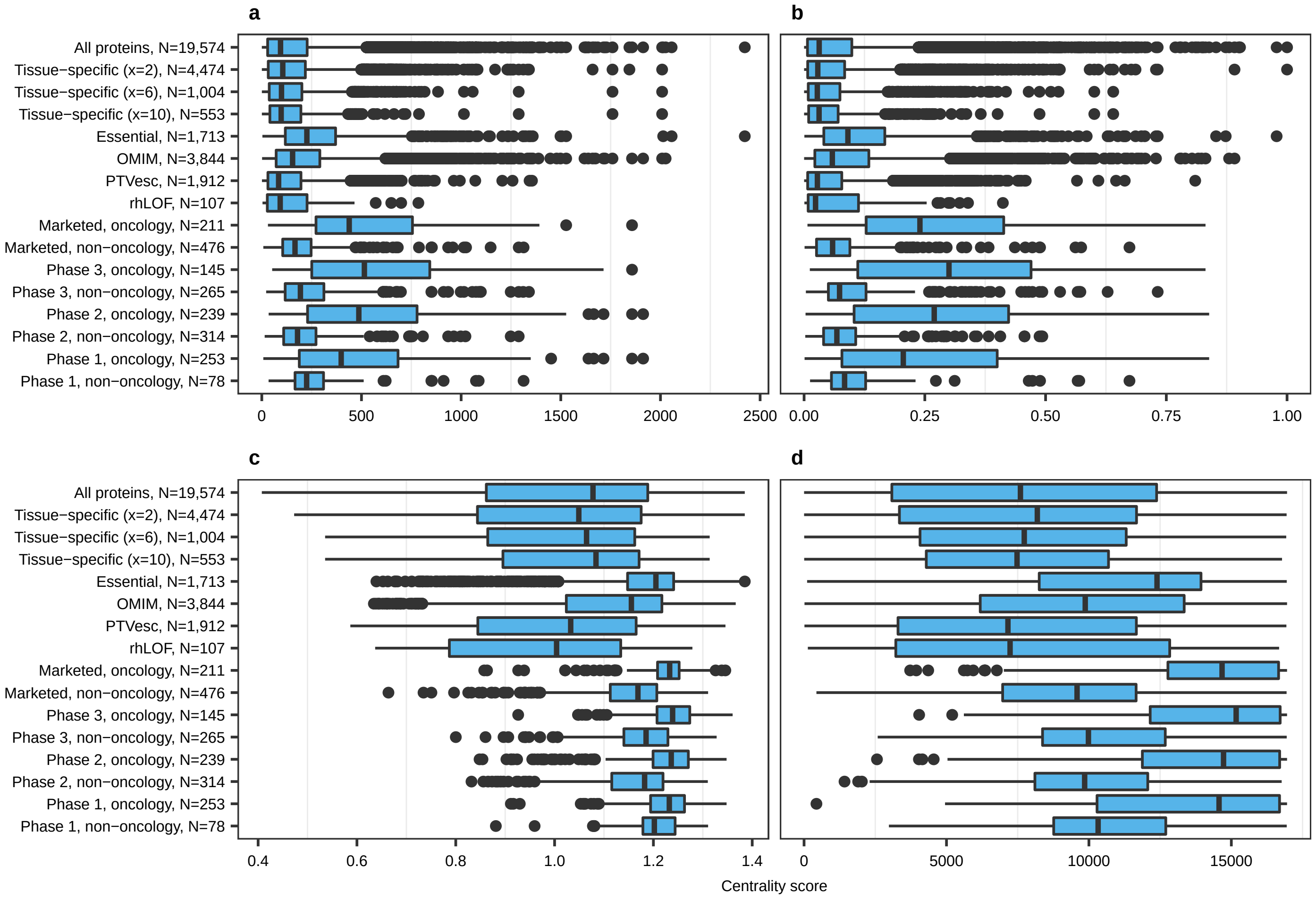

Supplementary Figure 10. Centrality scores in STRING v 10.5. **a** Strength. **b** Eigenvector centrality. **c** Closeness centrality (normalized). **d** Weighted k-shell. Discrepancies in sample size are due to different numbers of genes mapped between data sets.

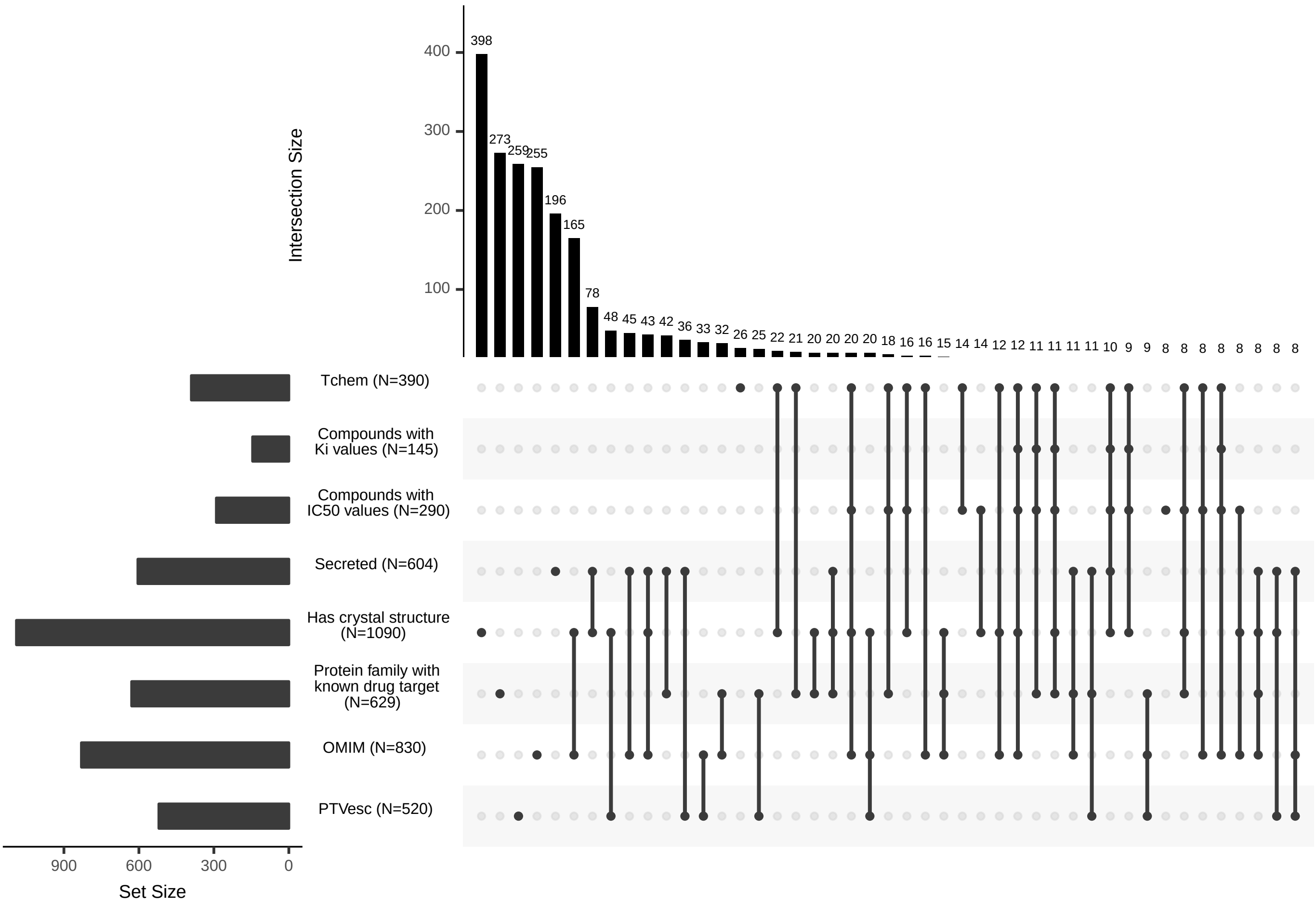

Supplementary Figure 11. Tissue-specific genes (x = 2) that were not yet explored as targets of marketed or clinical trial drugs but were potentially druggable or had human genetic evidence. Figure layout and notations are as in Fig. 5. Only the 45 largest set intersections are displayed to improve readability. In total, 4,305 were not explored as targets of marketed or clinical trial drugs, from which 1,587 were Tdark (36.9%), 1,986 (46.1%) had some indication of druggability and 705 (16.4%) had both some indication of druggability and human genetic evidence.

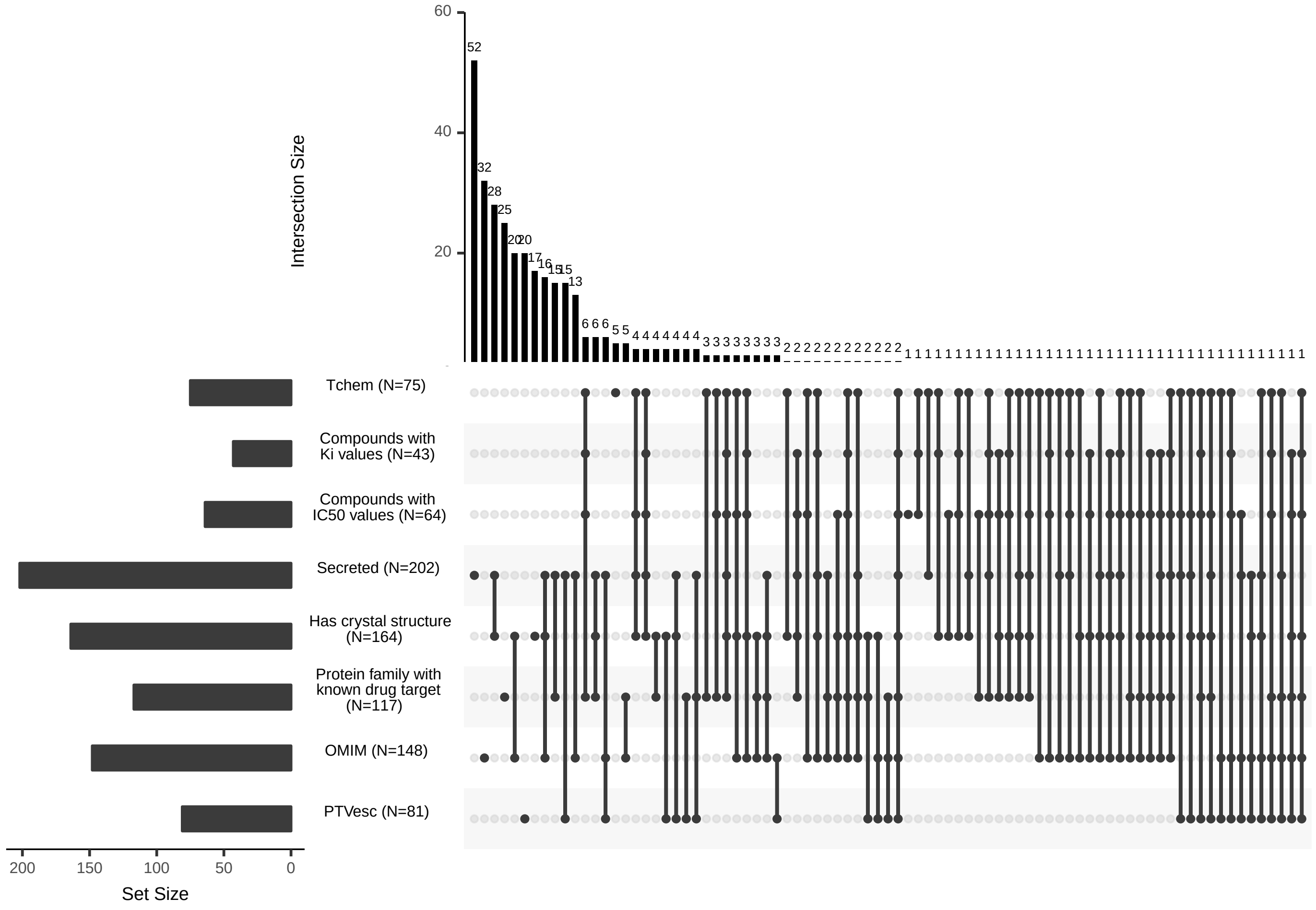

Supplementary Figure 12. Tissue-specific genes (x = 10) that were not yet explored as targets of marketed or clinical trial drugs but were potentially druggable or had human genetic evidence. Figure layout and notations are as in Fig. 5. In total, 489 were not explored as targets of marketed or clinical trial drugs, from which 91 were Tdark (18.6%), 342 (69.9%) had some indication of druggability and 150 (30.7%) had both some indication of druggability and human genetic evidence.
